# Gender and Family Disparities in Suicide Attempt and Role of Socioeconomic, School, and Health-Related Difficulties in Early Adolescence

**DOI:** 10.1155/2014/314521

**Published:** 2014-07-20

**Authors:** Kénora Chau, Bernard Kabuth, Nearkasen Chau

**Affiliations:** ^1^Service de Médecine Générale, Faculty of Medicine, Lorraine University, 9 Avenue de la Forêt de Haye, BP 184, 54505 Vandoeuvre-lès-Nancy Cedex, France; ^2^Service de Pédopsychiatrie, Hôpital d'Enfants de Nancy-Brabois, Faculty of Medicine, Lorraine University, 9 Avenue de la Forêt de Haye, BP 184, 54500 Vandoeuvre-lès-Nancy Cedex, France; ^3^INSERM, U669, Maison de Solenn, 97 Boulevard de Port Royal, 75679 Paris Cedex 14, France; ^4^University of Paris-Sud and University of Paris Descartes, UMR-S0669, Paris, France

## Abstract

Suicide attempt (SA) is common in early adolescence and the risk may differ between boys and girls in nonintact families partly because of socioeconomic, school, and health-related difficulties. This study explored the gender and family disparities and the role of these covariates. Questionnaires were completed by 1,559 middle-school adolescents from north-eastern France including sex, age, socioeconomic factors (family structure, nationality, parents' education, father's occupation, family income, and social support), grade repetition, depressive symptoms, sustained violence, sexual abuse, unhealthy behaviors (tobacco/alcohol/cannabis/hard drug use), SA, and their first occurrence over adolescent's life course. Data were analyzed using Cox regression models. SA affected 12.5% of girls and 7.2% of boys (*P* < 0.001). The girls living with parents divorced/separated, in reconstructed families, and with single parents had a 3-fold higher SA risk than those living in intact families. Over 63% of the risk was explained by socioeconomic, school, and health-related difficulties. No family disparities were observed among boys. Girls had a 1.74-time higher SA risk than boys, and 45% of the risk was explained by socioeconomic, school, and mental difficulties and violence. SA prevention should be performed in early adolescence and consider gender and family differences and the role of socioeconomic, school, and health-related difficulties.

## 1. Introduction

Every year, one million persons die from suicide worldwide [1]. Suicide is the 2nd cause of death among the persons aged 10–24 and represents 1.8% of total burden of diseases [1]. The suicide rate has increased leading youth to become most at risk for one-third of developed and developing countries [1]. France ranked third in the European Union in 2001 [1]. The suicide rate (per 100,000 subjects) strongly increases with age in youth: from 0.02 for the persons aged 5–9 to 7.8 for those aged 15–19 [2]. Suicide attempt (SA) is common among adolescents [3]. It is often a cry for help and attention and an expression of despair/wish to escape rather than a genuine intent to die [4–6]. SA can occur early (from 7 years) [7], and up to 23% of persons who consulted a physician for SA will relapse in the following year [8] and up to 10% of them will die from suicide during the five following years [9]. Further knowledge is needed for prevention. To date the causes of suicide remain partly documented [10]. A World Health Organization report based on recent research findings states that suicide is associated with a wide range of factors including mental illness, social isolation, substance abuse, and violence, but there are limited evidences for prevention approaches which need to address different risk factors at various risk levels [6]. Because girls have a much higher SA risk than boys [3] and because parents' separation/divorce and death can occur early (often before 6 years) and can then generate socioeconomic, school, and health-related difficulties [11], a question of interest is whether the risk of SA varies according to family structure in boys and girls, and what is the role of these difficulties in explaining the variations across family types and genders? Socioeconomic difficulties may be considered as initial causal factors while school and health-related difficulties as confounding or intermediate factors.

Indeed, during the last decades, families are greatly changing leading more children to have fewer siblings and to live with cohabiting, divorced/separated, or single parents [12]. Parents' separation/divorce often results in residence and living environment change, lower socioeconomic resources, and poorer social support. In France, the persons under poverty threshold represented 7.5% in 2009, 20.8% in single-parent families, and 43.8% in inactive mothers [13]. Over the past decade poverty in households with children is rising in nearly all OECD (Organisation for Economic Cooperation and Development) countries (12.7% across the OECD) [12]. Poor living conditions are well known to be associated with school difficulty, consumption of substance (alcohol, tobacco, cannabis, and hard drugs), mental disorders, and sustained violence [14–21]. These may favor SA because school difficulty reflects somewhat a mental/cognitive ability; depression affects physical and mental performances [22–25]; and sustained violence favors substance use, depressive symptoms, hopelessness, and altered cognitive development [3, 15–17, 20, 21]. Working and living difficulties favor substance use to cope [26, 27] and this substance use could alter in turn physical and mental performances [28–30] and could then increase living difficulties and SA risk. The knowledge of SA risk associated with gender and family structure and the role of socioeconomic, school, and health-related difficulties in early adolescence may help when designing SA prevention. Despite the abundant literature, the knowledge remains incomplete as studies have generally investigated few risk factors only and the time of occurring of SA as well as the exposure over time to various risk factors has often been unknown; most studies have been case-studies on hospital patients [3, 31–33].

In an early adolescence context in France, this study assessed the gender and family disparities in SA and the role of a wide range of covariates including socioeconomic factors, school difficulty, depressive symptoms, sustained violence, and unhealthy behaviors (tobacco, alcohol, cannabis, and hard drug use). We focused on early adolescence because these issues are common in this period [1, 3, 15, 16, 34, 35], most factors (except socioeconomic features) are modifiable for prevention, and various problems should be solved sooner (via early assessment, appropriate treatment, and monitoring). Early substance use is associated with a higher SA risk [3, 36]. As adolescents may be more impulsive and may focus more on proximal consequences of behavior than more distant goals when making decisions [37], we should help them to reduce sooner their problems which may lead to SA.

## 2. Materials and Methods

### 2.1. Procedure

The study population comprised all 1,666 students attending three middle schools, two public and one private, chosen as it may reflect a social gradient (various social categories are represented) in the urban area of Nancy (410,000 inhabitants), the capital of the Lorraine region (2,342,000 inhabitants) in north-eastern France. They cover a relatively large geographical area (comprising 38.000 inhabitants) and comprise 63 classes. The investigation was approved by the Nancy-Metz regional education authority and the Commission Nationale de l'Informatique et des Libertés (national review board). Written informed consent was obtained from the respondents.

The study protocol included an invitation to participate transmitted to parents/guardians (April 2010) and data were collected (May-June 2010) using an anonymous self-administered questionnaire filled in over a one-hour teaching period, under research-team supervision with teacher assistance (for surveillance, with no influence on the survey). Respondents were allowed to ask the two research-team members if they did not understand a question, but the team had been instructed not to say anything that might influence the response (they rarely did so). Completed questionnaires were put in sealed envelopes and then a closed box by the subjects. Two students refused to participate and 89 (5.3%) were absent when the data collection was carried out (for reasons independent of the survey). In total, 1575 subjects (95%) completed the questionnaires; 10 respondents were of unknown gender/age, and six questionnaires were not completed appropriately, leaving 1559 (94%) available for analysis. This population was close to that of a French school-based population survey in terms of gender, family, and health-related factors (Table 1).

The questionnaire included demographic and socioeconomic characteristics (birth month and year, gender, family structure, nationality, parents' education, father's occupation, family income, and social support), grade repetition, depressive symptoms, physical/verbal violence sustained by the adolescent, being victim of sexual abuse, unhealthy behaviors (alcohol, tobacco, cannabis, and hard drug use), SA, and the first occurrence/initiation of various life events via a historic reconstruction over the adolescent's life course.

### 2.2. Measures

SA was assessed with the question “During the life course, how many times did you actually attempt suicide?” (any/none) [3, 38]. The year of the first occurrence over the life course was also gathered.

For family structure, three categories were investigated: (a) intact families corresponded to the adolescents who were living with both nonseparated/nondivorced father and mother; (b) parents divorced/separated and reconstructed families corresponded to the adolescents who had parents separated/divorced with the presence or not of a father-in-law or a mother-in-law, and (c) single parent and other situations. Among the 391 subjects who were living with parents divorced/separated and in reconstructed families, 82.9%, 9.0%, 4.6%, and 3.6% were, respectively, living generally, sometimes, rarely, and never with mother (resp., 39.4%, 32.0%, 17.1%, and 11.5% with father). Because of small number of subjects which would result in a lack of power for statistical tests, the time spent with father and mother was not considered.

For father's occupation, five categories were considered following the international classification of occupations (ISCO): managers, professionals, and intermediate professionals; craftsmen, tradesmen, and heads of firms; service workers and clerks; manual workers and other occupations; and not working (unemployed and retired) [34, 39]. For perceived family income, subjects were asked whether the financial situation of their family was coping but with difficulties/getting into debt versus comfortable/well off/earning just enough [40]. Social support was measured with a 9-item scale: “During the last 12 months were you satisfied with support from your:” father, mother, father/mother-in-law, brothers/sisters, grand-parents, other family members, parents adoptive, host family, and friends (unsatisfied/indifferent versus satisfied/nonconcerned). Cronbach's alpha (a measure of reliability or internal consistency) was satisfactory (0.57), allowing a single score to be calculated as the number of positive responses (higher score represented lower social support). Social support was then categorized into 3 classes: 0, 1-2, and ≥3 (90th percentile).

Grade repetition was assessed with the question “During the life course, do you have repeated school year(s) at primary school and middle school?” (never, at primary school, for every year at middle school); multiple responses were possible [14]. The year of grade repetition(s) was gathered.

Depressive symptoms were measured with the Kandel scale [3, 41]. Cronbach's alpha was satisfactory (0.84) allowing a single score to be calculated (range 6–18). They were defined by a score ≥17 (90th percentile). The years of the first and last occurrences over the life course were gathered.

Physical/verbal violence sustained by adolescent was measured using a 20-item scale (five questions for four localities: in school, school neighborhood, at home, and elsewhere): “During the last 12 months, have you been victim of…?”: hitting, stealing, racket, insult, and racial abuse (any/none) [3, 38]. Cronbach's alpha was satisfactory (0.71), allowing a single score to be calculated as the number of positive responses. Sustained violence was defined by a score ≥4 (90th percentile). The years of the first and last occurrences over the life course were also gathered. Sexual abuse sustained was assessed with the question: “In the course of your life, have you been victim of a sexual abuse?” (any/none) [3, 38]. The years of the first and last occurrences were also gathered.

Use initiation of alcohol/tobacco/cannabis/hard drugs was assessed with the questions “During the life course,” “did you consume alcoholic drinks (beer, cider, champagne, wine, aperitif, etc.)?”, “did you smoke cigarettes?”, “did you used [sic] any form of cannabis?”, and “did you used [sic] any form of other illicit drugs (mushrooms, ecstasy, LSD, etc.)?” (any/none) [3, 34, 35, 38]. The year of initiation of each substance was also gathered.

A historic reconstruction of life events from birth to the day of survey was made using retrospective data. During the observation period, which represents 14,530 person-years (7,289 person-years for girls and 7,241 person-years for boys), 154 SA were observed (98 SA among girls and 56 SA among boys).

### 2.3. Statistical Analysis

Only the first SA was considered. The analyses were made for boys and girls separately. Three sets of covariates were investigated: socioeconomic factors (age, parents' education, occupation, nationality, family income, and social support), school and mental difficulties and sustained violence (grade repetition, depressive symptoms, sustained violence, and sexual abuse), and unhealthy behaviors (tobacco, alcohol, cannabis, and hard drug use). First, we assessed the differences between family types, for boys and girls separately, in terms of SA and various risk factors using the Chi^2^ tests. Then the risk of SA associated with various factors for adolescents living in each family type was assessed using Cox regression models to compute age-adjusted hazard ratios (ageHR) and 95% confidence intervals (CI). For each risk factor, the exposure period had begun from its first occurring to SA occurring or the day of survey. Adjustment for age was considered because age somewhat reflected an exposure duration for all life events. As SA could be seen as a generalization of survival process, the Kaplan-Meier survival estimates and log-rank test were also used to compare different family types. To evaluate the contributions of the three sets of covariates to the difference in SA risk between the adolescents living in nonintact families and those living in intact families, four Cox regression models were performed: a basic model (model 1) measuring the SA risk after adjustment for age, then socioeconomic factors added to model 1 (model 2), school and mental difficulties and sustained violence added to model 2 (model 3), and finally unhealthy behaviors added to model 3 (model 4). The contribution of each set of covariates was estimated by the change in the HRs, that is, explained fraction calculated by the formula (HR_model  1_ − HR_extended  model_)/(HR_model  1_ − 1) [42]. Finally, to assess the gender difference in SA and the roles of various sets of covariates, five Cox regression models were performed: a basic model (model 1) measuring the SA risk after adjustment for age, then family structure added to model 1 (model 2), socioeconomic factors added to model 2 (model 3), school and mental difficulties and sustained violence added to model 3 (model 4), and unhealthy behaviors added to model 4 (model 5). The analyses were performed using the Stata program (Stata Corporation, College Station, TX, USA, 2007).

## 3. Results

**Figure 1:**
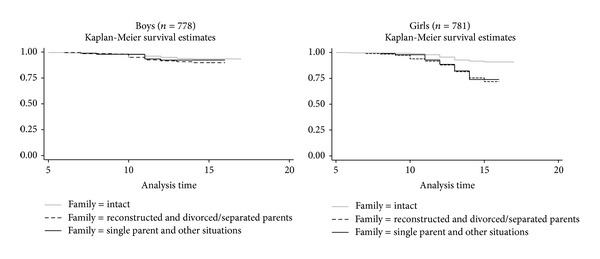
Frequency of subjects in various family structures with no suicide attempt according to age (year) among boys and girls. The log-rank test for equality of the “survivor functions” (for suicide attempt) was nonsignificant for boys (*P* = 0.279) and highly significant for girls (*P* < 0.0001).

SA affected 12.5% of girls and 7.2% of boys (*P* < 0.001). The mean age at first SA was 10.2 (SD 2.3) years for boys and 11.8 (SD 1.9) years for girls (*P* < 0.001). During the observation period from birth to the day of survey (14,530 person-years), 98 SA among girls and 56 SA among boys were observed.  Table 2 shows that the lifetime prevalence and the crude rate of SA (per 1,000 person-years) were 3-fold higher among girls living with parents divorced/separated, in reconstructed families or with single parents (crude rates 23.2 and 23.4 per 1,000 person-years) compared with those living in intact families (crude rate 7.8 per 1,000 person-years). These differences were not observed among boys. Similar results were found for multiple suicide attempts. These family disparities were observed since an early age (Figure 1). The mean adolescent's age at parents' separation/divorce and parent's death were, respectively, 6.2 (SD 3.9, range 0–16) and 7.7 (SD 3.9, range 0–14).  Table 2 further shows that living in non-intact families was associated with low parents' education, being immigrant, low father's occupation, insufficient family income, poor social support, grade repetition, depressive symptoms, and tobacco and cannabis use for both genders. Being victim of sexual abuse and alcohol and hard drug use were associated with living in nonintact families among girls only. The adolescents living with parents divorced/separated and in reconstructed families had the poorest social support for both genders.

In Table 3, we found that, among the adolescents living with parents divorced/separated and in reconstructed families, the associations between SA and poor social support, having sustained violence, sexual abuse, and cannabis use were significant for girls only (ageHR between 3.93 and 6.97). Among the adolescents living with single parents, the associations between SA and depressive symptoms, being victim of sexual abuse and tobacco, cannabis, and hard drug use were also significant for girls only (ageHR between 5.12 and 11.60). Furthermore, among the adolescents living with parents divorced/separated and in reconstructed families, the associations between SA and alcohol, tobacco, and hard drug use were stronger for girls (ageHR between 4.48 and 7.34) than for boys (between 3.66 and 5.53). Among the adolescents living in intact families, the associations between SA and grade repetition and alcohol use were significant for girls only (ageHR 2.43 and 3.85, resp.) whereas those between SA and social support score ≥3, depressive symptoms, having sustained violence, and tobacco use were stronger for girls (between 5.46 and 10.98) than for boys (between 3.21 and 5.53).

Table 4 reveals that the 3-fold higher SA risk for the girls living with parents divorced/separated and in reconstructed families and those living with single parents was greatly explained by socioeconomic factors (42% and 33%, resp.) and that adding school and mental difficulties and sustained violence to socioeconomic factors increased the contributions, respectively, to 60% and 37%, and further adding unhealthy behaviors increased the contributions, respectively, to 69% and 63%.

Table 5 shows that girls had a 1.74-fold higher SA risk than boys, which did not change when controlling for family structure (1.75) suggesting that the role of gender concealed that of family structure. The HR moderately decreased to 1.59 (contribution 20%) when controlling for socioeconomic factors. It highly decreased to 1.41 (contribution 45%) with further controlling for school and mental difficulties and sustained violence. Further controlling for unhealthy behaviors changed the HR to 1.55 (contribution 26%). It should be noted that grade repetition, depressive symptoms, having sustained violence, and being victim of sexual abuse were strongly associated with unhealthy behaviors and most of these associations were stronger among girls than boys (Table 6).

## 4. Discussion

The present original study sheds light on the risk patterns of suicide attempt for boys and girls living in nonintact families. Our results suggest that living in nonintact families is associated with a high risk of suicide attempt for girls only and that the risk is greatly explained by socioeconomic difficulties, school and mental difficulties, sustained violence, and unhealthy behaviors. Our results also suggest that the well-known higher risk of suicide attempt for girls is also strongly explained by these covariates. These findings are an additional piece to the literature and highlight the fact that prevention should consider the specific risk patterns for boys and girls and the role of study covariates.

First, our study shows that the girls living in nonintact families have a strongly higher risk of SA from an early age, but this was not observed for boys. It further shows that the higher risk for girls is strongly explained by socioeconomic difficulties. We found that parents' separation/divorce and death occurred early and the adolescents concerned had more socioeconomic vulnerabilities (low parents' education, being immigrant, low father's occupation, insufficient family income, and poor social support) and a higher risk of grade repetition, depressive symptoms, and tobacco and cannabis use. These difficulties were generally higher for girls than boys. Moreover, the girls (not boys) living in nonintact families also had a higher risk of sexual abuse and alcohol and hard drug use. So the girls in nonintact families appeared to be particularly vulnerable in terms of socioeconomic difficulties compared with their counterparts in intact families. We further found that the contribution of socioeconomic difficulties to SA risk was higher for the girls living with parents divorced/separated and in reconstructed families than for those living with single parents (42% and 33%, resp.). This difference may be partly explained by a poorer social support and its stronger association with SA. The consequences of separation, divorce, and death of parents would be thus higher among girls than among boys. This may be somewhat expected as girls have higher mental vulnerabilities [3]. These family issues often result in home and living environmental changes, school change, poorer social support, and lower financial resources. However, it may be noted that, in our study, poor social support was much less frequent among girls in intact families, but those who were affected also had a high SA risk. Social support played thus an important role for all adolescents.

Our study further demonstrates that school and mental difficulties and sustained violence have a high contribution to SA risk (in addition to socioeconomic factors) among the girls living in nonintact families and that the risk patterns greatly differ between the girls living with parents divorced/separated and in reconstructed families and those living with single parents. We found that the girls in the first group were more vulnerable for depressive symptoms while those in the second group were more vulnerable for sexual abuse. Having sustained violence was associated with SA among the girls in the first group whereas depressive symptoms and being victim of sexual abuse were more strongly associated with SA among the girls in the second group. It should be noted that the differences in SA risk with the girls living in intact families could be attributed to the higher frequency of school and mental difficulties and sustained violence as these factors were rather more strongly associated with SA among girls in intact families than among girls in nonintact ones. This result suggests that school and mental difficulties and sustained violence were less common among girls in intact families, but the girls affected were more subject to SA than those in nonintact families (maybe because of more mental vulnerability and isolation in a family context where such issues are less frequent [34, 43]). These results were expected because school difficulty reflects a mental ability, depression affects psychomotor and cognitive functions [22–25], while sustained violence favors mental disorders and alters cognitive development [3, 15–17, 20, 21]. Prevention to reduce SA should aim at limiting school and mental difficulties and sustained violence among the girls in nonintact families but also among those in intact families. These observations were also noted among boys. So such prevention could be conducted for both sexes.

Another important finding of our study is that unhealthy behaviors also strongly explain the higher SA risk (in addition to socioeconomic, school and mental difficulties, and sustained violence) for the girls living with parents divorced/separated and in reconstructed families and those living with single parents. In our study, all alcohol, tobacco, cannabis, and hard drug uses were much more common among the girls in the two types of nonintact families than among those in intact families. It may be noted that the differences between various family types were smaller for boys and significant for tobacco and cannabis use only. As girls may commit a SA more to call for help from the neighborhood while, for boys, SA represents a greater intention to die [4, 5] the higher substance use among girls may be partly seen as a help to cope with living and mental difficulties. Unfortunately, the substance use could alter in turn physical and cognitive capabilities and aggravate living and mental difficulties [28–30] leading to a higher SA risk. A SA results thus from a difficult life trajectory through family issues, socioeconomic, school and mental difficulties, sustained violence, and unhealthy behaviors which increase the vulnerability and the SA risk over time. Brent and Mann [44] reported the case of an adolescent aged 16 who committed a SA after the death of his father, followed by a depression, increasing alcohol use, and involvement as victim and perpetrator of rioting at school. Our results point out the complex risk patterns, the knowledge of which may be useful for physicians as well as for parents, school, and adolescents. The neighborhood may not be aware of the issues, especially because of their precociousness. Early adolescence corresponds to the mean age of onset of substance use, sleep disorders, sustained violence, and suicide behaviors [3, 34, 35, 38, 45, 46].

In line with the literature, we found that girls had a higher SA risk than boys [3]. We found that this gender difference concealed the family differences and that it was strongly explained by socioeconomic factors (20%) as well as by school and mental difficulties, and sustained violence (45%; the risk became nonsignificant after controlling for these two sets of factors). Interestingly, we found that adding unhealthy behaviors changed the contribution to SA risk to 26%. Our study shows that the SA risk magnitude was higher for girls than for boys for a wide range of covariates including especially poor social support, school and mental difficulties, sustained violence, and unhealthy behaviors. So, these factors may generate higher vulnerability for SA among girls. Compared with the adolescents in intact families, those in nonintact families consumed more substance probably to cope with living and mental difficulties, and the differences were more pronounced among girls than among boys. Prevention should thus consider specific issues and life trajectories of adolescents living in various family types. These results may help understanding the gender difference in SA risk and the parts played by a wide range of socioeconomic, school, and health-related difficulties. From a research perspective, studies on gender difference may include these potential factors. Our results are somewhat in agreement with those of other studies [3, 31–33, 47], but we investigated a wide range of covariates through a historic reconstruction over adolescents' life course.

Some methodological aspects warrant comments. The study was based on self-reported data, but this is widely used to study adolescent living conditions, mental health, and unhealthy behaviors [3, 34, 35, 38, 39]. Adolescents know their issues and well report them on self-administered questionnaire [5]. Study strengths included high participation rate (94%) and statistical approach based on life events historic reconstruction. However causal relationships could not be guaranteed because certain life events may be forgotten, but they were relatively recent and we think that the adolescents affected well remember them [35]. The interpretation of our results should be made with caution as the 95% CIs of HRs were often overlapped. Various measures were used in other studies [3, 30, 38, 41, 45, 48]. Grade repetition is an objective measure. The health and health-related behaviors of the sample were close to those of France. We did not investigate multiple suicide attempts because the time of occurring was available for the first suicide attempt only and because of a relatively small number of subjects to investigate the role of socioeconomic, school, and health-related difficulties in multiple suicide attempts.

## 5. Conclusion

This study demonstrates that, during early adolescence, which is crucial for youth development, the girls living with parents divorced/separated, in reconstructed families and with single parents have a 3-fold higher risk of suicide attempt. It fails to find such family disparities in SA risk among boys. It further shows that socioeconomic difficulties, school and mental difficulties, sustained violence, and unhealthy behaviors have high contributions to the SA risk for girls, and that the risk patterns associated with the covariates differ a lot between the girls living with parents divorced/separated and in reconstructed families and those living with single parents. These covariates also explain a great part of the gender difference in SA risk. Prevention strategies to reduce suicide attempt should focus on screening and monitoring school and health-related difficulties, especially among girls living in nonintact families with socioeconomic difficulties and poor social support, via physician-parent-school-adolescent collaborations. Our findings need however to be confirmed by further studies.

## Figures and Tables

**Table 1:** Comparison between the study population and France (ESPAD survey [3, 38, 45]: %.

	Study population (limited to <16 years^a^)	France (ESPAD survey)
		<16 years
Number of subjects	*1,524 *	*8,367 *
Last 12-month suicide ideation	11.6	9.1
Lifetime suicide attempt	9.6	7.2
Girls	50.1	51.1
Family structure		
Intact	63.2	74.7
Reconstructed	15.0	11.3
Single parent	16.4	11.7
Others	5.4	2.3
Obese (with self-reported data)	10.6	6.9
Last 30-day substance use		
Tobacco	10.7	13.6
Alcohol	34.7	34.6
Cannabis	5.1	5.5
Sleep disorders	32.6	29.0
Asthma	17.2	16.3
Depressive symptoms [41]	13.1	9.8
Being victim of sexual abuse	3.4	1.9
Sustained violence (at least once)	53.3	51.5
Involvement in violence (at least once)	59.1	64.7

^a^were excluded 35 subjects aged 16 years or over.

**Table 2:** Characteristics of male and female adolescents in various family structures: % or mean (SD).

	Boys				Girls			
	Intact families	Parents divorced/separated and reconstructed family	Single parent and other situations	Pearson's chi^2^ or Fisher's test (*P* value)	Intact families	Parents divorced/separated and reconstructed family	Single parent and other situations	Pearson's chi^2^ or Fisher's test (*P* value)
Number of subjects	*489 *	*199 *	*90 *		*493 *	*192 *	*96 *	
Number of person-years	*4,576 *	*1,836 *	*829 *		*4,629 *	*1,764 *	*896 *	
Lifetime suicide attempt(s)								
At least one	6.1	9.6	7.8	0.284	7.3	21.4	21.9	<0.001
Crude rate per 1,000 person-years	6.6	10.3	8.4		7.8	23.2	23.4	
Two or more	2.0	3.0	4.4	0.376	2.4	10.4	10.4	<0.001
Socioeconomic factors								
Age (year)								
Mean (SD)	13.45 (1.27)	13.40 (1.23)	13.39 (1.23)	0.856	13.44 (1.26)	13.60 (1.27)	13.74 (1.33)	0.0634
Range	10.4–16.9	10.5–16.4	10.8–16.3		9.9–16.9	11.2–16.2	10.9–18.7	
Low parents' education	44.6	55.3	63.3	<0.001	41.4	54.7	67.7	<0.001
Nationality				0.021				0.050
French	94.5	92.5	88.9		94.1	90.6	90.6	
European immigrants	2.5	5.0	2.2		3.8	4.7	2.1	
Non-European immigrants	3.1	2.5	8.9		2.0	4.7	7.3	
Father's occupation				<0.001				<0.001
Managers, professionals, and intermediate professionals	43.6	32.7	27.8		44.4	27.1	21.9	
Craftsmen, tradesmen, and firm heads	19.4	20.1	20.0		22.1	20.3	13.5	
Service workers and clerks	11.4	11.1	6.7		7.7	9.4	4.2	
Manual workers and other occupations	22.1	30.1	17.8		23.3	31.2	31.2	
Not working	3.5	6.0	27.8		2.4	12.0	29.2	
Insufficient family income	13.3	22.6	21.1	0.006	13.4	25.5	33.3	<0.001
Poor social support				<0.001				<0.001
Score								
0	56.0	37.2	45.6		50.3	25.5	35.4	
1-2	31.1	45.2	40.0		35.9	52.6	47.9	
≥3	12.8	17.6	14.4		13.8	21.9	16.7	
School and mental difficulties and sustained violence								
Grade repetition	12.1	19.6	21.1	0.010	9.3	21.9	26.0	<0.001
Depressive symptoms	5.9	12.6	5.6	0.009	15.8	28.6	16.7	<0.001
Having sustained violence	12.1	15.6	14.4	0.439	4.7	7.3	6.2	0.377
Being victim of sexual abuse	2.4	2.5	5.6	0.252	2.6	6.2	10.4	<0.001
Unhealthy behaviors								
Alcohol use	62.2	67.8	66.7	0.323	53.7	72.4	66.7	<0.001
Tobacco use	23.3	39.7	42.2	<0.001	19.3	41.2	45.8	<0.001
Cannabis use	7.8	16.1	14.4	0.003	3.8	7.3	14.6	<0.001
Hard drug use	4.9	8.0	8.9	0.157	1.6	6.8	10.4	<0.001

**Table 3:** Associations between suicide attempt (SA) and various risk factors for male and female adolescents in different family structures: age-adjusted hazard ratio (HR), 95% confidence interval (CI).

	Boys						Girls					
	Intact families		Parents divorced/separated and reconstructed family		Single parent and other situations		Intact families		Parents divorced/separated and reconstructed family		Single parent and other situations	
Number of person-years	*4,576 *		*1,836 *		*829 *		*4,629 *		*1,764 *		*896 *	
Socioeconomic factors												
Age (year)	0.91	0.68–1.23	0.81	0.57–1.24	1.03	0.57–1.89	1.10	0.82–1.47	0.93	0.70–1.22	1.25	0.86–1.82
Low parents' education	0.63	0.29–1.35	1.13	0.45–2.81	3.62	0.44–30.1	2.08∗	1.07–4.03	1.28	0.68–2.40	1.27	0.49–3.28
Nationality												
French	1.00		1.00		1.00		1.00		1.00		1.00	
European immigrants	1.47	0.20–10.9	2.37	0.54–10.3	0.00		2.25	0.69–7.35	1.61	0.50–5.24	2.45	0.33–18.4
Non-European immigrants	2.50	0.69–1.25	3.67	0.43–31.7	0.00		1.33	0.18–9.75	1.61	0.49–5.27	1.33	0.30–5.86
Father's occupation												
Managers, professionals, and intermediate professionals	1.00		1.00		1.00		1.00		1.00		1.00	
Craftsmen, tradesmen, and firm heads	1.66	0.67–4.14	1.63	0.41–6.53	2.91	0.26–32.8	0.74	0.29–1.87	1.40	0.56–3.54	3.46^§^	0.85–14.0
Service workers and clerks	1.04	0.29–3.72	2.38	0.53–10.7	0.00		0.96	0.28–3.28	1.50	0.50–4.51	0.00	
Manual workers and other occupations	1.29	0.50–3.34	2.04	0.59–7.01	3.33	0.30–37.3	1.08	0.50–2.37	0.95	0.38–2.34	1.51	0.38–6.06
Not working	1.22	0.16–9.44	1.41	0.16–12.6	2.02	0.17–23.7	0.00		2.05	0.77–5.42	1.19	0.28–5.00
Insufficient family income	2.55∗	1.13–5.76	2.04	0.80–5.22	1.56	0.27–9.05	2.42∗	1.17–5.02	1.46	0.75–2.82	1.72	0.73–4.08
Poor social support												
Score												
0	1.00		1.00		1.00		1.00		1.00		1.00	
1-2	4.25∗∗∗	1.75–10.3	2.69	0.42–3.97	1.55	0.33–7.22	4.44∗∗	1.63–12.1	**3.93** ∗	1.17–13.2	1.15	0.41–3.25
≥3	*4.67* ∗	1.62–13.5	2.69	0.82–8.80	0.00		*10.98* ∗∗	3.98–30.3	**6.97** ∗∗	1.98–24.5	2.16	0.69–6.71
School and mental difficulties and sustained violence												
Grade repetition	0.84	0.24–2.91	1.27	0.39–4.09	1.63	0.26–10.2	**2.43** ∗	1.04–5.64	1.82^§^	0.89–3.70	2.34^§^	0.91–6.03
Depressive symptoms	*3.35* ∗	1.28–8.76	4.84∗∗∗	1.89–12.4	3.65	0.39–34.3	*5.46* ∗∗∗	2.82–10.6	2.35∗∗	1.23–4.46	**5.12** ∗∗∗	2.17–12.1
Having sustained violence	*3.21* ∗∗	1.47–7.01	1.47	0.48–4.49	5.01∗	1.12–22.4	*5.73* ∗∗∗	2.50–13.1	**5.02** ∗∗∗	2.38–10.6	1.59	0.37–6.90
Being victim of sexual abuse	8.41∗∗∗	2.93–24.1	2.49	0.33–18.7	3.25	0.39–27.0	9.14∗∗∗	3.78–22.1	**3.46** ∗∗	1.44–8.32	**8.45** ∗∗∗	3.30–21.6
Unhealthy behaviors												
Alcohol use	1.72	0.76–3.94	*5.53* ∗	1.22–25.0	—^a^		**3.85** ∗∗	1.53–9.69	*7.34* ∗∗	1.75–30.7	3.65^§^	0.81–16.4
Tobacco use	*5.49* ∗∗∗	2.54–11.8	*4.09* ∗∗	1.50–11.2	1.84	0.38–8.87	*7.40* ∗∗∗	3.50–15.6	*7.13* ∗∗∗	3.19–16.0	**11.60** ∗∗∗	2.63–51.1
Cannabis use	4.48∗∗∗	1.84–10.9	2.30	0.80–6.56	0.95	0.11–8.20	2.59^§^	0.90–7.50	**5.86** ∗∗∗	2.82–12.2	**7.17** ∗∗∗	2.86–18.0
Hard drug use	7.41∗∗∗	3.13–17.5	*4.84* ∗∗	1.74–13.5	4.52^§^	0.87–23.3	7.61∗∗∗	2.59–22.4	*5.43* ∗∗∗	2.55–11.6	**6.02** ∗∗∗	2.42–15.0

*P < 0.05, ∗∗*P* < 0.01, ∗∗∗*P* < 0.001,^§^close to significance (*P* < 0.10).

^a^Noncomputable.

In bold types the HRs significant for girls but not for boys; in italic types the HRs significant for both genders but higher for girls.

**Table 4:** Suicide attempt risk associated with family structure and covariates contribution among girls (*n* = 781): adjusted hazard ratio (HR), 95% confidence interval (CI), and covariates contribution (%)^a^.

	Parents divorced/separated and reconstructed family (versus intact family)			Single parent and other situations (versus intact family)		
	HR	95% CI	%^a^	HR	95% CI	%^a^
Model 1: age-adjusted HR	**3.05** ∗∗∗	1.94–4.77	100	**2.98** ∗∗∗	1.74–5.11	100
Model 2: +socioeconomic factors^b^	**2.19** ∗∗∗	1.37–3.49	42	**2.33** ∗∗	1.31–4.13	33
Model 3: +school and mental difficulties and sustained violence^b^	**1.81** ∗	1.11–2.93	60	**2.25** ∗∗	1.27–3.99	37
Model 4: +unhealthy behaviors^b^	1.63	0.99–2.66	9	1.73	0.95–3.16	63

*P < 0.05, ∗∗*P* < 0.01, ∗∗∗*P* < 0.001. Bold types: significant HR.

^a^Covariates contribution (%) = reduction of (positive %) or increase (negative %) in HR computed with the following formula: (HR_model 1_ − HR_extended model_) /(HR_ model 1_ − 1).

^b^See Table 2.

Note: The age-adjusted HRs were nonsignificant for boys (1.58, 95% CI 0.89–2.80, and 1.28, 95% CI 0.56–2.92, respectively).

**Table 5:** Gender difference in suicide attempt and contributions of covariates (*n* = 1,559): adjusted hazard ratio (HR), 95% confidence interval (CI), and covariates contribution (%)^a^.

	Adjusted HR	95% CI	%^a^
Model 1: Age-adjusted HR	1.74∗∗∗	1.25–2.41	100
Model 2: +family structure	1.75∗∗∗	1.26–2.43	−1
Model 3: +other socioeconomic factors^b^	1.59∗∗	1.14–2.21	20
Model 4: +school and mental difficulties and sustained violence^b^	1.41	0.99–2.00	45
Model 5: +unhealthy behaviors^b^	1.55∗	1.08–2.22	26

*P < 0.05, ∗∗*P* < 0.01, ∗∗∗*P* < 0.001.

^a^% = Reduction of (positive %) or increase (negative %) in HR computed with the following formula: (HR_model 1_ − HR_extended model_)/(HR_ model 1_ − 1).

^b^See Table 2.

**Table 6:** Associations of unhealthy behaviors with school and mental difficulties and sustained violence among boys and girls: %.

	Boys (*n* = 778)				Girls (*n* = 781)			
	Alcohol use	Tobacco use	Cannabis use	Hard drug use	Alcohol use	Tobacco use	Cannabis use	Hard drug use
School and mental difficulties and sustained violence								
Grade repetition								
Absence	65.2	27.2	8.9	5.3	58.8	24.0	4.6	2.7
Presence	58.1	43.6	20.5	11.1	66.4	51.3	14.2	11.5
Relative risk	0.89	**1.60**	**2.30**	**2.09**	1.13	**2.13**	**3.09**	**4.26**
Chi^2^ test (P value)	*0.141 *	*<0.001 *	*<0.001 *	*0.016 *	*0.130 *	*<0.001 *	*<0.001 *	*<0.001 *
Depressive symptoms								
Absence	63.1	28.5	10.2	5.1	56.0	22.1	4.1	2.2
Presence	76.3	44.1	17.0	18.6	76.5	52.3	14.1	11.4
Relative risk	1.21	**1.55**	**1.66**	**3.65**	**1.36**	**2.37**	**3.44**	**5.18**
Chi^2^ test (P value)	*0.043 *	*0.012 *	*0.104 *	*<0.001 *	*<0.001 *	*<0.001 *	*<0.001 *	*<0.001 *
Having sustained violence								
Absence	61.9	26.7	9.3	4.6	59.1	26.7	5.1	3.2
Presence	78.6	49.5	19.4	16.5	74.4	48.8	20.9	16.3
Relative risk	1.27	1.85	**2.09**	**3.59**	1.26	1.82	**4.10**	**5.09**
Chi^2^ test (P value)	*<0.001 *	*<0.001 *	*0.002 *	*<0.001 *	*0.046 *	*0.002 *	*<0.001 *	*<0.001 *
Being victim of sexual abuse								
Absence	63.4	28.4	9.4	4.5	59.0	25.9	4.7	3.1
Presence	90.9	72.7	54.5	63.6	80.0	71.4	34.3	22.9
Relative risk	1.43	2.56	**5.80**	14.13	1.35	2.76	**7.30**	7.39
Chi^2^ test (P value)	*0.008 *	*<0.001 *	*<0.001 *	*<0.001 *	*0.013 *	*<0.001 *	*<0.001 *	*<0.001 *

Bold types: Relative risks higher for girls than for boys.
